# Methodological implications of sample size and extinction gradient on the robustness of fear conditioning across different analytic strategies

**DOI:** 10.1371/journal.pone.0268814

**Published:** 2022-05-24

**Authors:** Luke J. Ney, Patrick A. F. Laing, Trevor Steward, Daniel V. Zuj, Simon Dymond, Ben Harrison, Bronwyn Graham, Kim L. Felmingham

**Affiliations:** 1 School of Psychological Sciences, University of Tasmania, Tasmania, Australia; 2 Melbourne Neuropsychiatry Centre, Department of Psychiatry, University of Melbourne & Melbourne Health, Victoria, Australia; 3 School of Psychological Sciences, University of Melbourne, Victoria, Australia; 4 School of Psychology, Swansea University, Wales, United Kingdom; 5 Department of Psychology, Reykjavik University, Reykjavik, Iceland; 6 School of Psychology, University of New South Wales, New South Wales, Australia; Erasmus University Rotterdam: Erasmus Universiteit Rotterdam, GERMANY

## Abstract

Fear conditioning paradigms are critical to understanding anxiety-related disorders, but studies use an inconsistent array of methods to quantify the same underlying learning process. We previously demonstrated that selection of trials from different stages of experimental phases and inconsistent use of average compared to trial-by-trial analysis can deliver significantly divergent outcomes, regardless of whether the data is analysed with extinction as a single effect, as a learning process over the course of the experiment, or in relation to acquisition learning. Since small sample sizes are attributed as sources of poor replicability in psychological science, in this study we aimed to investigate if changes in sample size influences the divergences that occur when different kinds of fear conditioning analyses are used. We analysed a large data set of fear acquisition and extinction learning (N = 379), measured via skin conductance responses (SCRs), which was resampled with replacement to create a wide range of bootstrapped databases (*N* = 30, *N* = 60, *N* = 120, *N* = 180, *N* = 240, *N* = 360, *N* = 480, *N* = 600, *N* = 720, *N* = 840, *N* = 960, *N* = 1080, *N* = 1200, *N* = 1500, *N* = 1750, *N* = 2000) and tested whether use of different analyses continued to produce deviating outcomes. We found that sample size did not significantly influence the effects of inconsistent analytic strategy when no group-level effect was included but found strategy-dependent effects when group-level effects were simulated. These findings suggest that confounds incurred by inconsistent analyses remain stable in the face of sample size variation, but only under specific circumstances with overall robustness strongly hinging on the relationship between experimental design and choice of analyses. This supports the view that such variations reflect a more fundamental confound in psychological science—the measurement of a single process by multiple methods.

## Introduction

Fear conditioning paradigms are critical to understanding and improving treatment for several psychiatric disorders, including post-traumatic stress disorder (PTSD) and anxiety [1, 2]. Fear extinction occurs when a previously conditioned fear stimulus (conditioned stimulus, CS+) is repeatedly presented without aversive reinforcement, causing new safety information to compete with pre-existing fear memory [3–5]. Patients with anxiety-related disorders show deficits in extinction learning, which is believed to facilitate disease progression and maintenance [6, 7]. The rate of an individual’s fear extinction learning can be estimated by the decrease in threat response to the unreinforced CS+ when compared to the safety signal (CS-), typically indexed via various physiological measures [8], with skin conductance responses (SCRs) being most commonly used. Extinction learning has been subject to extensive research on its neurobiological basis [9–19], and serves as a highly informative framework for investigating pharmacological and psychological adjuncts to exposure therapy for PTSD, and deficits associated with treatment outcomes [20–27].

The replicability crisis has inspired a growing movement dedicated to improving the quality of research practices in psychological science [28–32]. These issues of replicability extend to research on human fear conditioning [8]. Importantly, inconsistent research practices in fear conditioning might explain the contradictory and null outcomes identified across recent large-scale studies and meta-analyses [7, 33–36]. These limitations have been identified for several methodological domains including, but not limited to, study design [37, 38], pre-processing of psychophysiological data [39–42], and statistical analysis strategies [43–46]. It is increasingly clear that issues such as these undermine the replicability of fear conditioning research, and the subsequent translation of experimental findings to clinical outcomes.

Our previous report [54] was concerned with the effect of analytic strategy on robustness. Simply put, ‘robustness’ in psychology refers to the ability for a result to be consistent across multiple arbitrary statistical specifications [28]. In our case, arbitrary specifications associated with inconsistency in analytical strategies and we demonstrated stark divergence of effect sizes when different statistical methods were used to index extinction [47]. Specifically, a large data set was resampled to create 40 data sets of *N* = 60 rows with three groups per sample. Different statistical strategies, all intending to measure extinction, were compared against each other across the 40 data sets, but varied with respect to the numbers of trials included the stages of the phases the trials were drawn from, and whether the data was analysed trial-by-trial or averaged. We tested the effect of these variations on robustness of studies that compared acquisition learning to extinction learning, change in responding during extinction (e.g., early to late extinction), and where extinction was treated as a single effect estimate. We showed that the rank order of these strategies varied significantly depending on the data set, which illustrates less than desirable robustness of these statistical tests [47]. However, solutions to the issue of inconsistent analytic strategy remain unexplored.

In the current study, we aimed to investigate one plausible solution—increased sample size. Increasing sample size will increase the power of a study—that is, the ability to detect a specific effect size within a sample. It has been observed that many fear conditioning studies may be underpowered due to low samples [39] and it is possible that improving the precision of physiological measures through more advanced pre-processing could be sufficient to improve robustness of fear conditioning and extinction outcomes [39–41, 48]. By increasing power, we increase the probability of detecting the effect, and it is possible that heterogeneity of outcomes can be caused by underpowered studies that do not accurately capture this effect. However, heterogeneous statistical analyses have been reported to produce misleading or false results independent of power considerations [28, 49].

To investigate if larger samples could address the analytical issue we previously identified, we bootstrapped data from existing data sets, obtaining rank orderings of previously used statistical methods for indexing fear acquisition and extinction [47]. In our previous study, each resampled data set had a sample size of *N* = 60 rows, broken into three groups during analysis. Here, we resampled from our real data (*N* = 379) with replacement to create bootstrapped samples of *N* = 30, 60, 120, 180, 240, 360, 480, 600, 720, 840, 960, 1080, 1200, 1500, 1750, and 2000 observations, with each row being equivalent to one subject. These numbers were chosen to cover a broad range of plausible sample sizes used in human fear conditioning research. We performed two experiments—in the first, group allocation was randomised, and no group-level effect was anticipated. In the second, we added a group-level effect to our bootstrapped data. The group level effect was varied across three conditions, which were roughly based on [50] with one group who had high responding during acquisition and rapid extinction, another group who had lower responding during acquisition and rapid extinction, and another group who did not extinguish the CS+ response. This work represents a significant contribution above our previous study because (a) we create much larger samples spanning a wide range of simulated sample sizes; and (b) we test the results of this and our previous work against the presence of a simulated group-level effect. Testing our hypotheses with the inclusion of simulated group-level effects is a significant contribution because most fear conditioning studies will observe group differences and our original analyses were likely not representative of these studies; hence, the effect of heterogeneity of analytical methods in studies with groups effects is unknown. In this extension of our previous work, we therefore aimed to identify possible boundary conditions of an originally bleak report of the robustness of statistical analysis pipelines for fear conditioning research.

We hypothesised that larger samples would not improve robustness of rank ordering between analytic strategies in either condition, because we believe that the issue of analytical heterogeneity is a fundamental violation of replicability that cannot be solved by increasing power alone. We hypothesised that the type of simulated effect would vary the robustness of different statistical strategies because some strategies are used to examine different stages of learning during fear conditioning tasks.

## Methods

The current manuscript uses secondary data analysis strategies on existing datasets, and did not require further ethical approval. The original studies received ethical approval from the University of Tasmania Social Sciences Human Research Ethics Committee. The fear acquisition and extinction procedures, as well the data set, for this study are identical to those of our previous study [47]. Briefly, six data sets gathered over seven years were resampled with replacement to form new samples. Participants reported no significant physical illnesses, no history of head trauma or loss of consciousness, no current or significant historical use of illicit substances, and no heavy alcohol use or dependence. Of the 379 participants included in this dataset, *N* = 51 (13.46%) had a diagnosis of PTSD (clinician diagnosed) or had a score above 40 on the PCL-IV or above 30 on the PCL-5 [51, 52]. No other psychiatric diagnoses were permitted in any of the studies. PTSD cases were retained in the sample in order to remain consistent with the previous study [47]. Since the predictor variable in these studies are the analytical strategies themselves, it is unlikely that systemic variability in participant characteristics would affect results [47].

### Fear conditioning paradigm and equipment

As in our previous report [47], data was obtained from five trials of acquisition learning and ten trials of extinction learning (split into early and late extinction phases of five trials each, which were separated by an instruction screen) across a total of 379 participants across the six studies. Acquisition and extinction phases were also separated by an instruction screen, which in all cases read “In the following phase, you may or may not receive shocks. Please press any key to continue”. For each trial, a CS+ (a coloured circle) and a CS- (a different coloured circle) were presented on a computer screen for 12s with intertrial intervals of 12-21s (*M* = 16s). In all studies, skin conductance was recorded from the first and third fingers of the left hand in micro-Siemens (μS) using a 22 mVrms, 75 Hz constant-voltage coupler (ADInstruments). A stimulus isolator (ADInstruments) was placed on the right hand and delivered a 500ms electric shock immediately following the CS+ offset during acquisition learning. No shocks were delivered during the extinction learning phase. Skin conductance responses (SCRs) were scored using a custom-coded peak scoring method which subtracts the average skin level 2s prior to CS onset from the peak conductance occurring 0.9-5s following CS onset, which scores the first interval response, and it should be noted that studies score skin conductance responding differently [53]. A bidirectional Butterworth filter was applied to the raw SCR trace to reduce noise.

### Resampling procedure

Data was bootstrapped (i.e., resampled with replacement) using rows of participant data [54]. Using bootstrapping, it is possible to validate the accuracy of statistical techniques across a range of sample sizes, and this has been done in previous literature assessing the effect of sample size on correlation, factor analysis, principal components analysis, prognostic modelling, and other statistical techniques [55–58]. Data was resampled by row such that all CS+ or CS- responses from a particular phase (e.g., acquisition) were resampled together. New data sets of *N* = 30, *N* = 60, *N* = 120, *N* = 180, *N* = 240, *N* = 360, *N* = 480, *N* = 600, *N* = 720, *N* = 840, *N* = 960, *N* = 1080, *N* = 1200, *N* = 1500, *N* = 1750, and *N* = 2000 rows were created and a ‘Group’ variable consisting of equal but random allocation of belonging to the number 1, 2, or 3. Therefore, no group-level effects were expected in this analysis. Sample sizes were chosen to cover a wide range of possible study power in the simulated datasets. These sample sizes were determined arbitrarily due to current debate concerning accurate power determination of fear conditioning research using skin conductance responding [39]. Three groups were used because in our field of research (PTSD) it is typical to examine a PTSD group against both a trauma-exposed control and a non-trauma exposed control group [59].

**Fig 1:**
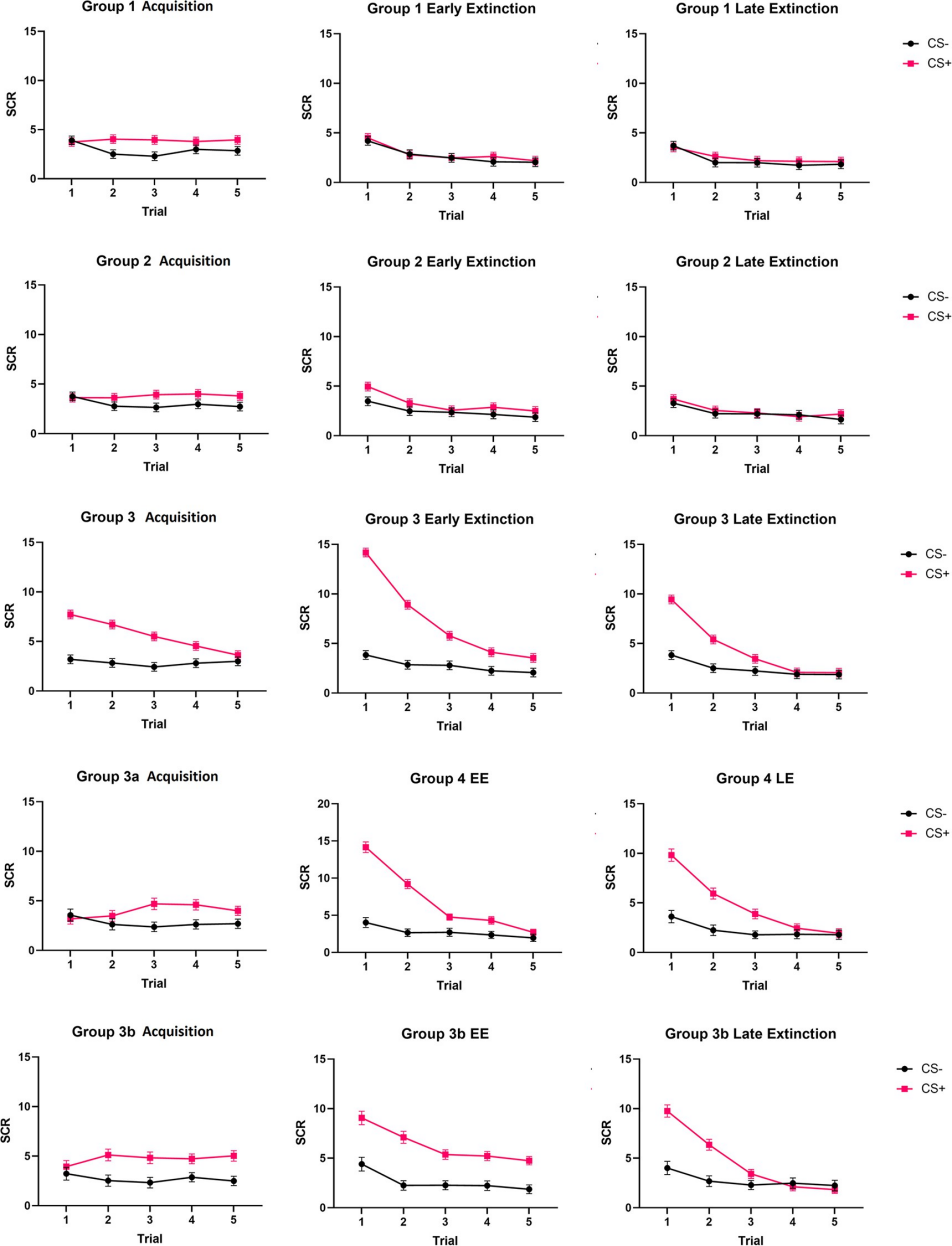
Example of simulated group-level effects in bootstrapped data (N = 960). SCR = Skin conductance response. Groups 3-3b have simulated effects of differing gradients to reflect possible differences in physiological expression of acquisition and extinction between participants and between studies. Error bars are 95% Confidence Intervals.

For the second experiment, scores were modified for the third Group upon bootstrapping such that a higher but gradually decreasing CS+ response (relative to CS- response) was expected in each phase. Data was produced that resembled the three fear conditioning trajectories reported by [50]. These trajectories were replicated in our own clinical fear conditioning data (manuscript in preparation), and group-level simulated effects were created in the data from the current report based on the difference between each of the three trajectories and our bootstrapped data that did not have a simulated group-level effect. The modifications to produce the simulated effects are described below. Scores for Group 3’s CS+ were modified to be 1, 0.8, 0.6, 0.4 and 0 standard deviations higher than their bootstrapped values during trials 1–5 of acquisition; 2, 1.5, 1, 0.8, 0.5 standard deviations higher than their bootstrapped values during trials 1–5 of early extinction; and 1, 0.8, 0.5, 0.2, and 0 standard deviations higher than their bootstrapped values during trials 1–5 of late extinction. Two other distinct group-level effects were simulated for Group 3, with CS+ modified to be 0, 0.3, 0.3, 0.3, and 0.3 standard deviations higher during acquisition, 1, 1, 1, 1, and 1 standard deviations higher during early extinction, and 1.5, 1, 0.5, 0.1, and 0 standard deviations during late extinction higher than the average data for Group 3a; and 0, 0, 0.3, 0.3, and 0.3 standard deviations higher during acquisition, 2, 1.5, 1, 0.5, and 0.3 standard deviations higher during early extinction, and 1.5, 1, 0.5, 0.1, and 0 standard deviations during late extinction higher than the average data for Group 3b. These simulated effects were achieved by adding the same value (e.g., 2 standard deviations above the mean score for trial 1) to all scores individually within that group. Therefore, all analyses in this study were conducted three times with Group 3 consisting of one of the three sets of simulated effects. An illustration of an example of this data is provided in Fig 1. Simulated effects were roughly based on the findings of Galatzer-Levy et al. (2017) [56], who identified three distinct trajectories during acquisition and extinction phases in fear conditioning data. In Fig 1, Group 3 is the trajectory that shows high differential acquisition and rapid extinction, Group 3a is the trajectory that shows moderate differential acquisition and rapid extinction, and Group 3b is the group that does not show extinction.

### Types of analytical strategies included in comparisons

Further analyses were conducted using base R. Analytic strategies were identical to those used previously [47] and are summarised in Table 1. As described previously, some strategies averaged trials or subtracted CS- from CS+ scores, whereas others did not. These details are described in Table 1. The goal of these strategies was to either: (1) determine the change in SCRs from acquisition to extinction learning (CON-EXT); or (2) determine a static measure of extinction learning (EXT) or (3) determine the change in SCRs across the extinction learning phase (EXT-EXT). Since the goals of the strategies differed in these ways, we divided strategies into each of these categories and compared outcomes only within each category.

**Table 1:** Description of different strategies for measuring extinction learning using skin conductance responses (Ney et al., 2020).

Analytic strategy	Strategy #	# of Trials	Trials Included	Trial Analysis	Stimuli Analysis	Analysis	Study
**ACQ—EXT**	Strategy 1	8 (ACQ), 16 (EXT)	All (ACQ), last 2 (EXT)	Average	Diff	Phase×group	[60]
	Strategy 2	5 (ACQ), 10 (EXT)	Maximum Response (ACQ), Last 2 (EXT)	Average	Diff	Phase×group	[61]
	Strategy 3	8 (ACQ), 7 (EXT)	All (ACQ), last 3 (EXT)	Average	Diff	Phase×group	[62]
	Strategy 4	20 (ACQ), 20 (EXT)	Last half (ACQ), First half (EXT)	Average, using paired t-test contrasts^	Diff	Phase×group	[63]
**EXT**	Strategy 1	16	Last three-quarters	Average	CS+, CS-	Group×stim	[64]
	Strategy 2	5	All	Trial-by-trial	CS+, CS-	Trial×Group×Stim	[65]
	Strategy 3	16	Last half	Average	CS+, CS-	Group×stim	[66]
	Strategy 4	10	Last trial	One trial	Diff	Group	[67]
	Strategy 5	10	Last 2	Average	CS+, CS-	Group×stim	[68]
	Strategy 6	5	All	Running average^#^	Diff	Trial×Group	[69]
	Strategy 7	8	First 2	Trial-by-trial	Diff	Trial×Group	
**EXT**_**early**_ **-EXT**_**late**_	Strategy 1	6	First half, second half	Average	CS+, CS-	Phase×Group×Stim	[70]
	Strategy 2	14	First half, second half	Average	Diff	Phase×Group	[71, 72]
	Strategy 3	16	First quarter, last quarter	Average	CS+	Phase×Group	[73]
	Strategy 4	32, 16	First half, second half	Average	CS+	Phase×Group	[74, 75]

ACQ = Acquisition, EXT = Extinction, Diff = Differential, CS+ = Conditioned stimulus to the aversive unconditioned stimulus, CS- = Conditioned stimulus as a safety signal, Stim = stimulus type (CS+ v. CS-).

^This study was the only study to use a test other than ANOVA.

^#^Running average response was calculated with trials one and two averaged as a single response, trials two and three averaged, and so on.

### Data analysis

For each strategy, we compared the highest order group-level interaction via its computed partial eta squared (*ɳp*^*2*^) effect size. For each sample size, bootstrapped (1,000 times) Kendall non-parametric ranked order correlation coefficients (_*T*_*b*) and associated 95% bootstrapped confidence intervals were computed between analytical strategies of each of the three categories, based on the *ɳp*^*2*^ effect sizes generated. Therefore, each sample size (e.g. 30 “participants”) was resampled 100 times to generate a rank order (_*T*_*b*) of *ɳp*^*2*^ across the different analysis strategies, and this procedure was bootstrapped 1,000 times to generate mean _*T*_*b* and 95% confidence intervals. The mean _*T*_*b* and its associated confidence intervals were the average correlation between one strategy and each of the other strategies separately (e.g., creating three mean _*T*_*b* values for Strategy 1 of the acquisition—extinction category). This entire procedure was completed using a custom R script that is available from the authors upon request. The data was compiled and is reported in the Supplementary Material up until *N* = 960. Data beyond this size is not reported due to excessive amount of the data reported in the manuscript and because the results at *N*>960 were almost identical to those obtained at *N* = 960. Using the average _*T*_*b* effect size of each strategy, we tested whether the rank order coefficients improved with increased sample size using Pearson’s coefficient (*r*). This was completed for both the first (no group-level effect) and second (simulated group-level effect) experiments.

During data compilation, it was evident that there were large decreases in effect sizes with increased sample size (*p* < .001). As an exploratory analysis, effect sizes averaged across sample sizes for each category of analytical strategy were compared using Pearson’s correlations (*r*). To ensure that the effects observed in this exploratory test were not due to variability caused by our resampling process (where CS+ or CS- scores for each participant from only one phase were resampled), we resampled using the full data from each participant to create data sets of *N* = 30, *N* = 60, *N* = 120, *N* = 240 rows, with three equally sized groups randomly allocated amongst these rows. Samples were not created that were larger than the number of actual participants to avoid repeating participant data in the same sample. Again, the *ɳp*^*2*^ effect sizes from each category of analytical strategy were averaged and compared across sample size.

## Results

**Fig 2:**
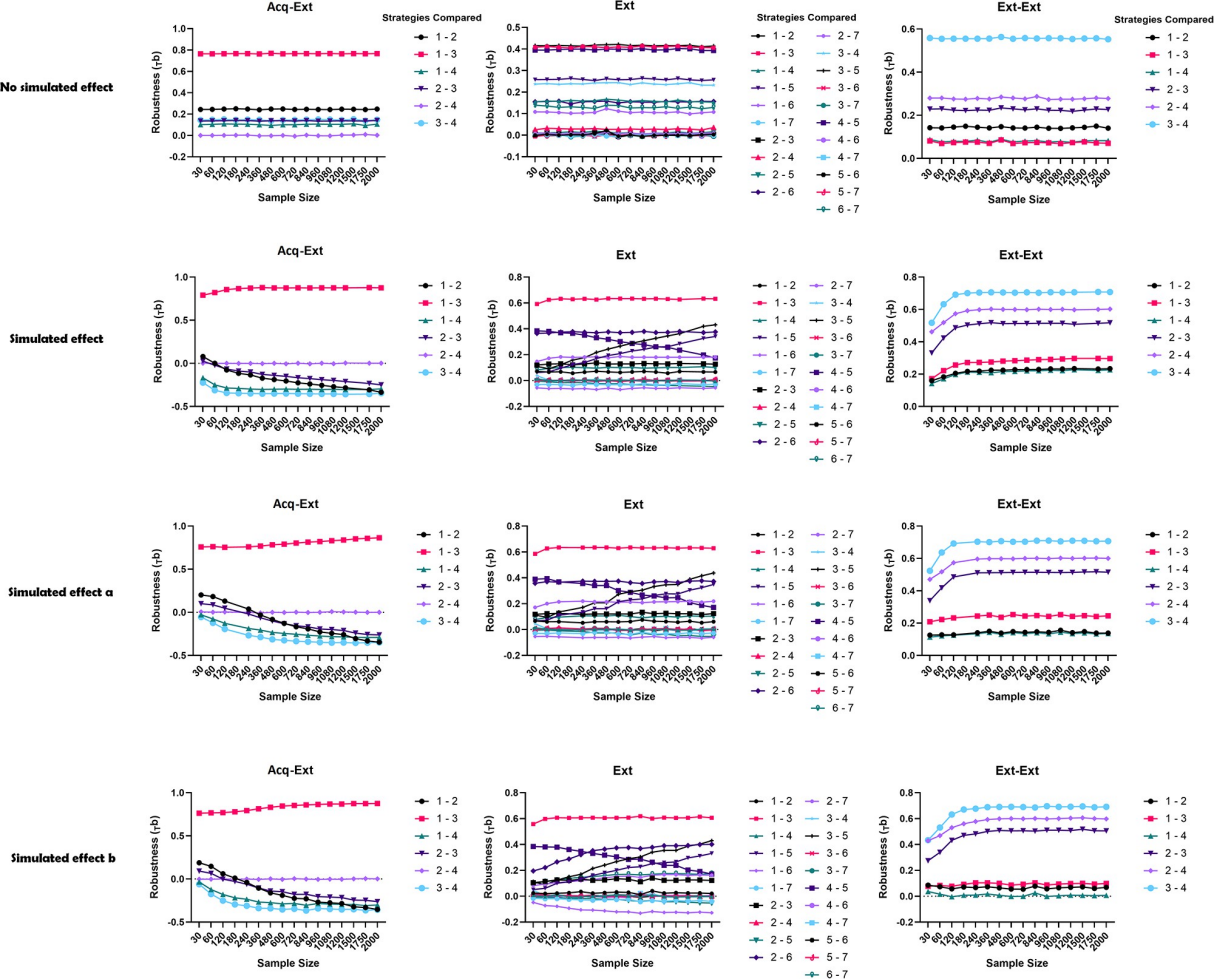
Effect of sample size on average Kendall’s rank order effect size (_*Τ*_*b*) between statistical strategies attempting to elicit the same construct from different data sets. Higher _*Τ*_*b* implies higher robustness. Top panel is data without simulated group-level effect, second panel simulates rapid decreasing differential conditioning during acquisition, third panel simulated gradual decrease in differential conditioning during acquisition, fourth panel simulated no change in differential conditioning during acquisition or early extinction.

The overall data from the original sample (*N* = 379) is reported in S1 Fig. The main index that was used as an outcome in the present study was the rank order of effect sizes produced by different analytical (i.e., statistical) approaches when applied to the same dataset. To ensure that this result was robust, datasets were bootstrapped so that the analysis was repeated many times. If a low rank order effect is produced, this implies that application of different analytical approaches to the same datasets produces inconsistent effect sizes relative to the other approaches. A high rank order effect suggests that application of different approaches to the same datasets produces consistent effect sizes relative to the other approaches, which implies robustness. To assess the robustness of each analytical method within each bootstrapped dataset, Kendall’s rank correlation coefficient values (_*T*_*b*) and corresponding 95% confidence intervals were computed for each of the three sets of analyses with sample size set to *N* = 30, *N* = 60, *N* = 120, *N* = 180, *N* = 240, *N* = 360, *N* = 480, *N* = 600, *N* = 720, *N* = 840, *N* = 960, *N* = 1080, *N* = 1200, *N* = 1500, *N* = 1750, and *N* = 2000 rows. Complete statistics from an exemplar of these analyses are reported in S1–S42 Tables and are summarised in Fig 2. We also entered the rank order for each analytical strategy compared to every other strategy into Pearson correlation models across each sample size. This data is visualised in Fig 2 and reported in Table 2.

**Table 2:** Pearson’s correlation coefficient and significance of the relationship between sample size and rank order between different statistical strategies used to index static extinction (EXT), change in extinction (EXT-EXT) and acquisition to extinction (ACQ-EXT) during fear learning paradigms.

**Strategies Compared (EXT)**																						
		1–2	1–3	1–4	1–5	1–6	1–7	2–3	2–4	2–5	2–6	2–7	3–4	3–5	3–6	3–7	4–5	4–6	4–7	5–6	5–7	6–7
**Strategies Compared (ACQ-EXT, EXT-EXT)**																						
		1–2	1–3	1–4	2–3	2–4	3–4															
**No simulated effect**																						
**ACQ-EXT**	*r*	-.096	.221	-.023	.063	.348	-.030															
	*p*	.723	.411	.933	.817	.187	.913															
**EXT**	*r*	-.150	-.060	-.396	-.220	.050	.010	-.157	.087	-.031	.171	-.166	-.356	-.325	-.009	-.282	-.188	.049	-.589*	.229	-.069	-.467
	*p*	.579	.825	.128	.414	.855	.971	.561	.749	.910	.528	.539	.176	.219	.972	.290	.485	.858	.016	.394	.800	.068
**EXT-EXT**	*r*	-.071	-.265	-.028	-.088	-.078	-.209															
	*p*	.794	.321	.917	.746	.775	.437															
**Simulated effect**																						
**ACQ-EXT**	*r*	-.869**	.529*	-.455	-.901**	.146	-.431															
	*p*	< .001	.043	.089	< .001	.602	.109															
**EXT**	*r*	-.032	.391	-.507	.955**	.346	.217	.234	-.043	-.947**	.331	.353	-.359	.961**	.215	.034	-.990**	-.010	.094	-.104	.189	.515*
	*p*	.910	.150	.054	< .001	.206	.438	.400	.880	< .001	.228	.197	.188	< .001	.442	.905	< .001	.971	.740	.712	.500	.049
**EXT-EXT**	*r*	.661**	.662**	.641*	.482	.499	.448															
	*p*	.007	.007	.010	.069	.058	.094															
**Simulated effect a**																						
**ACQ-EXT**	*r*	-.932**	.981**	-.810**	-.919**	.009	-.736**															
	*p*	< .001	< .001	< .001	< .001	.976	.002															
**EXT**	*r*	-.077	.275	-.288	.964**	-.427	-.082	.057	-.543*	-.931**	.293	.458	-.375	.968**	-.177	-.217	-.986**	-.180	-.132	-.026	.047	.339
	*p*	.786	.321	.298	< .001	.112	.771	.839	.037	< .001	.289	.086	.169	< .001	.527	.437	< .001	.521	.640	.928	.869	.216
**EXT-EXT**	*r*	.407	.496	.482	.540*	.562*	.492															
	*p*	.132	.060	.069	.038	.029	.062															
**Simulated effect b**																						
**ACQ-EXT**	*r*	-.892**	.884**	-.697**	-.893**	.405	-.622*															
	*p*	< .001	< .001	.003	< .001	.120	.010															
**EXT**	*r*	-.078	.402	-.326	.958**	-.747**	-.777**	.203	-.433	-.969**	.794**	.833**	-.429	.958**	-.416	-.071	-.989**	.417	.057	-.289	-.167	.777**
	*p*	.773	.122	.218	< .001	.001	< .001	.451	.094	< .001	< .001	< .001	.098	< .001	.109	.795	< .001	.108	.833	.277	.537	< .001
**EXT-EXT**	*r*	-.204	.412	-.282	.587*	.619*	.531*															
	*p*	.449	.113	.290	.017	.011	.034															

Note: EXT = Static Extinction, ACQ-EXT = acquisition to extinction, EXT-EXT = early to late extinction.

*p < .05,

**p < .001

Overall, findings for non-simulated effect datasets are congruent with our previous findings [54], which was conducted with a sample size of *N* = 60. There were no significant trends in the data for the no-effect data (summarised in Table 2), which suggests that increasing sample size did not improve robustness caused by variability in analytical strategies used to assess similar constructs in the same data.

### ACQ-EXT

Strategies 1 and 3, which compared acquisition to extinction, produced high correlative values across sample sizes, whereas Strategies 2 and 4 were not similar to any Strategies (S22–S28 Tables). This finding replicated our findings from our previous report at *N* = 60.

When a group-level effect was simulated, however, these results changed. Only Strategies 1 and 3 showed positive but increasingly weak correlative improvements in the acquisition with increasing sample size (Fig 2 and S1–S7 Tables), whereas combinations of other strategies were increasingly significantly and negatively correlated with increased sample sizes, meaning that they estimated fear responding in opposite directions to one another and that this pattern got worse with a larger sample (Fig 2 and Table 2).

### EXT

Some of the correlations between the static extinction strategies failed to be supported compared to our previous study in the data without simulated group level effects (S29–S35 Tables). These were mainly between strategies 1, 4 and 5, which were not supported in data derived from the new data sets but had been correlated in our previous report. Correlations between Strategies 1 and 3; 2, 6 and 7; and 5, 3, and 4 continued to be supported of the static extinction strategies (S29–S35 Tables and Fig 2).

There were very few supported correlations in static extinction Strategies when group effects were simulated (i.e., high _*T*_*b* values, primarily correlations between 1 and 3 were supported), but some of these improved with increased sample size (Fig 2, Table 2, and S8–S14 Tables). Correlations between Strategies 1, 3, and 5 for static extinction improved significantly with increased sample size, whereas correlations between Strategies 2, 4, and 5 were significantly negatively correlated with increasing sample size. Other combinations of strategies showed no change with increasing sample size (Fig 2 and Table 2).

### EXT-EXT

At higher sample sizes, some of the significant correlations from our earlier study [47] within the early-late extinction strategies were no longer significant, though this did not follow a particularly consistent pattern (S40–S42 Tables). In all cases, Strategies 3 and 4 of the early-late extinction category continued to be correlated (Fig 2 and S36–S42 Tables), but this did not improve with higher sample size (Table 2).

Strategies 2 and 4, 3 and 4, as well as 2 and 3 of early-late extinction showed some moderate-high evidence of correlation that improved logarithmically when group level effects were simulated (Fig 2 and S15–S21 Tables). Unexpectedly, these results were not substantially affected by the type of simulated effect (Fig 2). However, only Strategies correlating with Strategy 1 from early-late extinction changed by improving with increased sample size, after correcting for multiple comparisons using False Discovery Rate Q = .1.

### Sample size and average effect sizes

**Fig 3:**
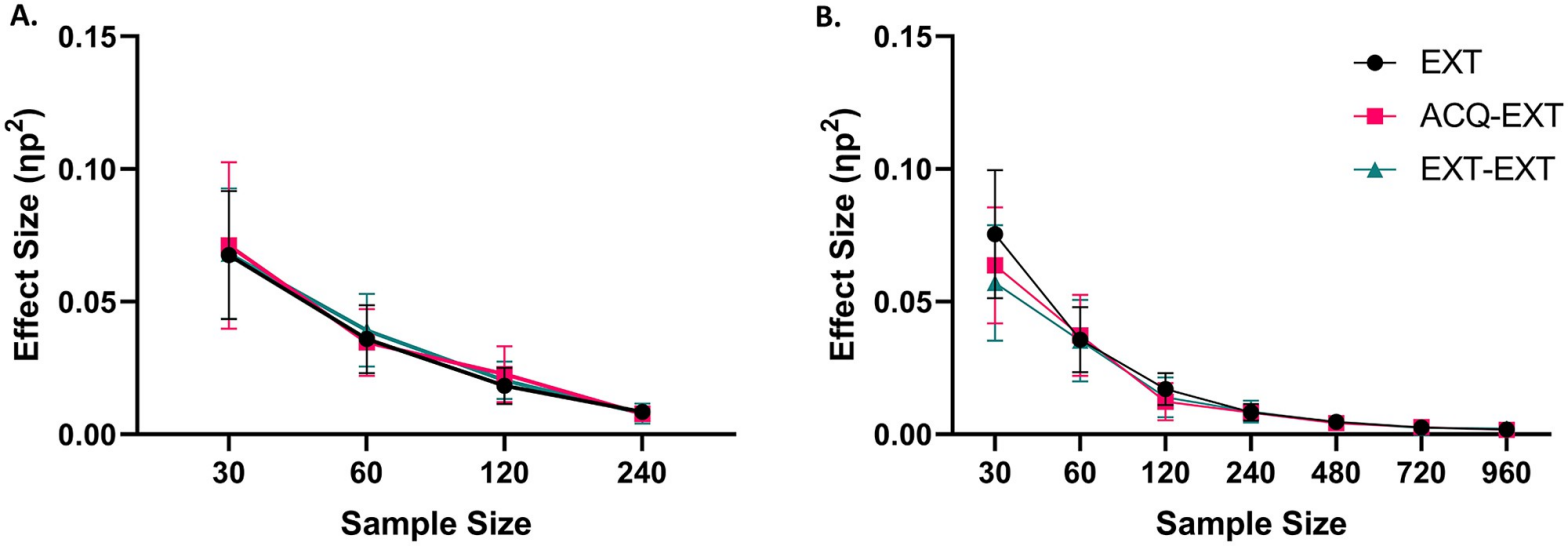
Average effect size decreased as sample size increased for all types of analyses (*p* < .001). Panel A is the correlation using resampled data of responses. Panel B is the correlation using resampled data of responses. Error bars are 95% Confidence Intervals.

During data compilation, we noticed large decreases in effect sizes with increased sample size. As an exploratory analysis, we correlated the average effect size (*ɳp*^*2*^) from each category of analytical strategies with the sample size. The average effect size for each set of analyses decreased significantly as a function of sample size (all *p* < .001), as shown by Fig 3A. Effect sizes of all three types of analyses reduced at a similar rate. This effect was replicated when the data was resampled from full participant rows (i.e., in real data, Fig 3B).

## Discussion

In this study we investigated whether the decreased robustness that arises from inconsistent analytic strategy [54] could be amended by increased sample sizes. To do so, we tested whether greater sample sizes affected the robustness of outcomes via lower divergence of results obtained across varied analytical strategies. Robustness did not improve when sample size was increased for any of the strategies included in our analysis that did not include a simulated effect. However, in contrast to our hypothesis, a simulated effect resulted in several changes in robustness, particularly within strategies that examined extinction as a single index. The kind of effect that was simulated (in terms of the gradient of fear responding across trials) did not substantially affect these results. These findings have several implications for study design and statistical analysis of fear extinction via SCRs.

Our previous study provided evidence that heterogeneity in analytical strategy in the assessment of fear extinction can reduce robustness of effects when tested across different data sets [47]. This problem has been reported in other fields, such as human neuroimaging [76, 77], and high flexibility in data analysis is an established cause of increased false positives [49]. It is possible, however, that some types of strategies produce more robust results than others. The current findings support several assertions that we made in our previous paper in this regard. First, studies that examine change in SCRs from acquisition to extinction will show varying robustness depending on what sections of acquisition and extinction are used, but robustness does not seem to be affected if small variations in number of trials or use of averaged compared to maximal values are used. Similarly, analysing extinction on a trial-by-trial basis is inconsistent with strategies that averaged across trials, but both strategies are internally consistent regardless of the number of trials included, or whether differential responses (CS+ > CS-) were calculated. Finally, we found mixed evidence that number of trials and use of differential responding affects robustness of strategies examining change during extinction, which were associations that we had previously identified as having moderate support [47].

The main aim of the current study was to understand whether improving power, by increasing sample size, would improve robustness that is affected by heterogeneity of analytical strategies. As we had anticipated, the limitations imposed by varied analytic strategies holds even when applied to samples of greater size, but this appeared to apply only when no effect was present in the data. While this supports the validity of our prior study [54], it also challenges our previous findings in several ways. Firstly, in the data containing simulated group-level effects, some strategies improved markedly in terms of robustness as sample size increased. However, these cases were contradicted by several other strategies that showed weaker robustness with increasing sample size. Most importantly, not all strategies showed these patterns, and marked improvement in robustness in the static-extinction strategies was primarily observed at the higher sample sizes, which research groups would not have the capacity to collect. Improving robustness of strategies examining changes in fear responding from early to late extinction might be achievable by increasing sample size to an amount that is viable with respect to research resources. Critically, these results were not substantially affected by alteration of the gradient of the group-level effect. This implies that it is possible that an improved set of data analysis strategies for fear extinction data could be applied robustly across fear extinction phenotypes, which were recently identified in [50]. These findings provide critical boundary conditions and caveats to our previous findings, and we strongly emphasise that not all analytical approaches that we highlighted as problematic (or robust) in our previous report will be applicable to all real-world samples. Critically, by extension our current data also demonstrates that a statistical approach that seems unrobust in this study could be robust if the underlying effect is different.

This research is important because sample considerations are frequently the first criticism addressed in experimental psychology research, and supports the notion that further methodological innovations are required to enhance fear extinction research, beyond simply increasing study power [43]. Several research groups have begun moving towards Bayesian inference in fear extinction [78–81] and computational modelling has also been explored in assessing physiological responses to fear conditioning [45]. It is possible that these contemporary statistical frameworks may offer solutions to the deficits imposed by heterogeneous analytic strategies in extinction research. However, further research is needed to explore this as a viable possibility to conventional data analysis, particularly in terms of accessibility to non-statisticians.

While compiling the data in the current study, we observed strong effects of lower sample size resulting in higher effect size. It is likely that the reduced effect size we observed with increasing sample size reflected increasing precision of the effect size, which is reflected in the increasingly narrow confidence intervals. The relationship between smaller samples reaching significance with higher effect sizes is intrinsically wedded to the parameters of power analysis in null-hypothesis significance testing (NHST) [82]. In a simple case, when performing *a-priori* power analysis (to determine an appropriate sample size), specifying higher *r* (e.g., effect size) and a significance criterion (α) of *p* < 0.05 will result in a generally lower *N*, all things being equal [83]. The propensity for studies with small sample sizes to inflate effect sizes is well documented [84–86]. This is sometimes attributed to publication bias [85], but in the context of the current study, higher variability in our smaller samples is a likely cause, as indicated by 95% confidence intervals. Our findings suggest that these issues are likely to be prevalent until a minimum of *n* = 40 participants per group for a 3-group design (which may vary depending on the number of groups). However, it has been reported that this estimate is improved by advanced SCR scoring methods [39]. Interestingly, the inflection point of logarithmic improvement in some of the strategies in terms of robustness was at this same sample size, raising the possibility that there may be some relationship between adequately powered data and the propensity for certain strategies (mainly the early-late extinction strategies) to perform robustly. Relatedly, power analyses using single point estimates from previous fear conditioning studies is likely problematic given that heterogeneity of experimental parameters and effect sizes that are chosen by researchers affect power calculations. Instead, it might be more useful to estimate the expected variability and build a power analysis based on the precision of the anticipated effect size (i.e., the effect size’s confidence interval) [87].

Considering this finding, as well as the overall results of the current report, we suggest two implications for the enhancement of robustness in fear extinction research via SCR. First, in line with our prior report (Ney et al., 2020), it is critical that a specific analytic strategy is implemented only when the experimenter seeks to measure a specific aspect of fear extinction, one that corresponds clearly to the strategy in question. For instance, some of the analytic strategies identified in this and the prior study [54] can credibly be used to measure distinct aspects of extinction learning. For instance, subtracting early and late extinction responses might represent a principled measure of extinction learning per se, while subtracting mean extinction responses from mean acquisition responses could represent something quite different, albeit equally worthy of investigation. Critically, if these different strategies are used, it is incumbent on the experimenter to interpret the results consistently. Labelling all different strategies under a homogenised term (i.e., ‘extinction learning’) could otherwise incur costs to robustness, and ultimately, failures to replicate. Similarly, it is important that standardised methods for comparing extinction between group relative to acquisition learning are developed, because there is significant heterogeneity in current methods that do this [46], yet some relative estimation is essential given that the effects observed during extinction are often contingent on responses during acquisition.

Second, this study illustrates that the pervasive issue of measuring one construct by a diverse array of analyses remains an issue even in the face of some methodological changes, in this case, sample size. An implication of this is that other methodological changes may also be unable to ameliorate this effect, but more critically, that future research should strive to find ways to analysis extinction learning that circumvent the effect altogether. In other words, analysing data in different ways will almost always lead to different outcomes, and reduced robustness or replicability. Therefore, rather than finding ways to homogenise between different analytic strategies as a path forward, ongoing work could seek to characterise extinction via more principled quantitative approaches. It is critical to consider that fear acquisition and extinction are multifaceted processes that cannot be captured by a single parameter. In many cases, researchers will make different statistical decisions based on the type of learning process that they are interested in—for example, analysing data trial-by-trial may assess the rate of learning, whereas comparing mean responses during extinction to acquisition might assess someone’s relative performance between phases. One way of addressing the propensity for different studies to use different types of analyses is to use multiverse approaches. Multiverse analysis is an approach that assesses a statistical problem with multiple analytical methods [88]. In fear conditioning, multiverse packages have been written for R [89], and can potentially directly address the issues highlighted within this paper by increasing transparency of statistical decision making as well as the relative importance of a reported result [53]. In this way, not only does multiverse analysis reduce the potential of p-hacking, but it also facilitates comparison between studies that may have otherwise analysed their results in incomparable ways. Similar to this, it is almost certain based on this and recent data that different experimental designs (e.g., number of trials, induction of uncertainty via instructions, etc) are likely to produce different outcomes that may not be readily comparable between studies. We are aware of current work aiming to produce ‘typical’ fear conditioning experiments that may help to standardise the field, but in the meantime it is also possible that further investigation of the relationship between specific statistical analyses and experimental designs may help to improve the comparability of findings between fear conditioning studies.

The current study is primarily limited by the possibility of our findings not generalising to other fear extinction designs. For instance, we have a relatively low number of trials and long-duration stimuli (12 s), which are not the case for many studies. Further, these results may not be transferable to different data pre-processing methods and will need to be checked independently by groups that use these methods. One issue that we did not explicitly examine was the effect of number of trials on statistical outcomes—however, it is probable that the number of trials included in a study presents another significant heterogeneity factor that, when analysed using similar methods, may reduce robustness. Our experimental phases were all separated by brief instruction screens, including between early and late extinction learning, and this detail may have impacted on the patterns observed in our results. Third, our sample included a small proportion of PTSD participants, though this was done to replicate our previous study [47]. While we do not anticipate that this would affect our primary outcome, some variability in the bootstrapped samples may have been due to participant characteristics such as this. Next, we only simulated one type of potential group-level effect in our data and this may have resulted in some strategies showing greater or lesser robustness, depending on the aim of the strategy. Therefore, we cannot be prescriptive concerning which strategy may perform best with group-level effects; however, it is relevant to note that a model that best describes extinction has not been formalised and thus it is unknown what group-level extinction data should look like. Finally, there may be many more analytical strategies in the literature that were not included in the present paper. These strategies could alter the robustness between strategies reported here. The strategies reported here were identical to those identified in the previous paper—based on highly cited examples; hence, it is possible that there are different analytical strategies reported in less cited studies.

In conclusion, we found that larger sample size does not improve the robustness of fear extinction results when assessed across heterogeneous analytical strategies when no effect is simulated but does alter robustness under some circumstances when an effect is simulated. We also report that smaller sample sizes (less than *N* = 120, or *n* = 40 per group) result in inflated effect sizes, both in simulated and original data. Although this issue is not unique to fear extinction, formal identification of it may encourage better powered studies and more progressive methods in the future. Future studies should examine how robustness of fear extinction analyses can be improved and ensure that studies are adequately powered such that effect sizes are not artificially inflated.

## Supporting information

S1 Table Conditioning—Extinction, N = 30. Strategy comparisons using Kendall rank correlation coefficient between effect-simulated datasets with changes from Conditioning to extinction learning phases estimated.
(DOCX)

S2 Table Conditioning—Extinction, N = 60. Strategy comparisons using Kendall rank correlation coefficient between effect-simulated datasets with changes from Conditioning to extinction learning phases estimated.
(DOCX)

S3 Table Conditioning—Extinction, N = 120. Strategy comparisons using Kendall rank correlation coefficient between effect-simulated datasets with changes from Conditioning to extinction learning phases estimated.
(DOCX)

S4 Table Conditioning—Extinction, N = 240. Strategy comparisons using Kendall rank correlation coefficient between effect-simulated datasets with changes from Conditioning to extinction learning phases estimated.
(DOCX)

S5 Table Conditioning—Extinction, N = 480. Strategy comparisons using Kendall rank correlation coefficient between effect-simulated datasets with changes from Conditioning to extinction learning phases estimated.
(DOCX)

S6 Table Conditioning—Extinction, N = 720. Strategy comparisons using Kendall rank correlation coefficient between effect-simulated datasets with changes from Conditioning to extinction learning phases estimated.
(DOCX)

S7 Table Conditioning—Extinction, N = 960. Strategy comparisons using Kendall rank correlation coefficient between effect-simulated datasets with changes from Conditioning to extinction learning phases estimated.
(DOCX)

S8 Table Static Extinction, N = 30. Strategy comparisons using Kendall rank correlation coefficient between effect-simulated datasets with a static extinction learning efficacy estimated.
(DOCX)

S9 Table Static Extinction, N = 60. Strategy comparisons using Kendall rank correlation coefficient between effect-simulated datasets with a static extinction learning efficacy estimated.
(DOCX)

S10 Table Static Extinction, N = 120. Strategy comparisons using Kendall rank correlation coefficient between effect-simulated datasets with a static extinction learning efficacy estimated.
(DOCX)

S11 Table Static Extinction, N = 240. Strategy comparisons using Kendall rank correlation coefficient between effect-simulated datasets with a static extinction learning efficacy estimated.
(DOCX)

S12 Table Static Extinction, N = 480. Strategy comparisons using Kendall rank correlation coefficient between effect-simulated datasets with a static extinction learning efficacy estimated.
(DOCX)

S13 Table Static Extinction, N = 720. Strategy comparisons using Kendall rank correlation coefficient between effect-simulated datasets with a static extinction learning efficacy estimated.
(DOCX)

S14 Table Static Extinction, N = 960. Strategy comparisons using Kendall rank correlation coefficient between effect-simulated datasets with a static extinction learning efficacy estimated.
(DOCX)

S15 Table Early—Late Extinction, N = 30. Strategy comparisons using Kendall rank correlation coefficient between effect-simulated datasets with changes during extinction learning estimated.
(DOCX)

S16 Table Early—Late Extinction, N = 60. Strategy comparisons using Kendall rank correlation coefficient between effect-simulated datasets with changes during extinction learning estimated.
(DOCX)

S17 Table Early—Late Extinction, N = 120. Strategy comparisons using Kendall rank correlation coefficient between effect-simulated datasets with changes during extinction learning estimated.
(DOCX)

S18 Table Early—Late Extinction, N = 240. Strategy comparisons using Kendall rank correlation coefficient between effect-simulated datasets with changes during extinction learning estimated.
(DOCX)

S19 Table Early—Late Extinction, N = 480. Strategy comparisons using Kendall rank correlation coefficient between effect-simulated datasets with changes during extinction learning estimated.
(DOCX)

S20 Table Early—Late Extinction, N = 720. Strategy comparisons using Kendall rank correlation coefficient between effect-simulated datasets with changes during extinction learning estimated.
(DOCX)

S21 Table Early—Late Extinction, N = 960. Strategy comparisons using Kendall rank correlation coefficient between effect-simulated datasets with changes during extinction learning estimated.
(DOCX)

S22 Table Conditioning—Extinction, N = 30. Strategy comparisons using Kendall rank correlation coefficient between datasets with changes from Conditioning to extinction learning phases estimated.
(DOCX)

S23 Table Conditioning—Extinction, N = 60. Strategy comparisons using Kendall rank correlation coefficient between datasets with changes from Conditioning to extinction learning phases estimated.
(DOCX)

S24 Table Conditioning—Extinction, N = 120. Strategy comparisons using Kendall rank correlation coefficient between datasets with changes from Conditioning to extinction learning phases estimated.
(DOCX)

S25 Table Conditioning—Extinction, N = 240. Strategy comparisons using Kendall rank correlation coefficient between datasets with changes from Conditioning to extinction learning phases estimated.
(DOCX)

S26 Table Conditioning—Extinction, N = 480. Strategy comparisons using Kendall rank correlation coefficient between datasets with changes from Conditioning to extinction learning phases estimated.
(DOCX)

S27 Table Conditioning—Extinction, N = 720. Strategy comparisons using Kendall rank correlation coefficient between datasets with changes from Conditioning to extinction learning phases estimated.
(DOCX)

S28 Table Conditioning—Extinction, N = 960. Strategy comparisons using Kendall rank correlation coefficient between datasets with changes from Conditioning to extinction learning phases estimated.
(DOCX)

S29 Table Static Extinction, N = 30. Strategy comparisons using Kendall rank correlation coefficient between datasets with a static extinction learning efficacy estimated.
(DOCX)

S30 Table Static Extinction, N = 60. Strategy comparisons using Kendall rank correlation coefficient between datasets with a static extinction learning efficacy estimated.
(DOCX)

S31 Table Static Extinction, N = 120. Strategy comparisons using Kendall rank correlation coefficient between datasets with a static extinction learning efficacy estimated.
(DOCX)

S32 Table Static Extinction, N = 240. Strategy comparisons using Kendall rank correlation coefficient between datasets with a static extinction learning efficacy estimated.
(DOCX)

S33 Table Static Extinction, N = 480. Strategy comparisons using Kendall rank correlation coefficient between datasets with a static extinction learning efficacy estimated.
(DOCX)

S34 Table Static Extinction, N = 720. Strategy comparisons using Kendall rank correlation coefficient between datasets with a static extinction learning efficacy estimated.
(DOCX)

S35 Table Static Extinction, N = 960. Strategy comparisons using Kendall rank correlation coefficient between datasets with a static extinction learning efficacy estimated.
(DOCX)

S36 Table Early—Late Extinction, N = 30. Strategy comparisons using Kendall rank correlation coefficient between datasets with changes during extinction learning estimated.
(DOCX)

S37 Table Early—Late Extinction, N = 60. Strategy comparisons using Kendall rank correlation coefficient between datasets with changes during extinction learning estimated.
(DOCX)

S38 Table Early—Late Extinction, N = 120. Strategy comparisons using Kendall rank correlation coefficient between datasets with changes during extinction learning estimated.
(DOCX)

S39 Table Early—Late Extinction, N = 240. Strategy comparisons using Kendall rank correlation coefficient between datasets with changes during extinction learning estimated.
(DOCX)

S40 Table Early—Late Extinction, N = 480. Strategy comparisons using Kendall rank correlation coefficient between datasets with changes during extinction learning estimated.
(DOCX)

S41 Table Early—Late Extinction, N = 720. Strategy comparisons using Kendall rank correlation coefficient between datasets with changes during extinction learning estimated.
(DOCX)

S42 Table Early—Late Extinction, N = 960. Strategy comparisons using Kendall rank correlation coefficient between datasets with changes during extinction learning estimated.
(DOCX)

S1 Fig Overall responding in the real data set. (TIF)
