# Supplementary material for: Methodological implications of sample size and extinction gradient on the robustness of fear conditioning across different analytic strategies
Source: PLoS One. 2022 May 24;17(5):e0268814. doi: 10.1371/journal.pone.0268814 (PMC9128987; doi:10.1371/journal.pone.0268814)
Supplement: S1 Table — Strategy comparisons using Kendall rank correlation coefficient between effect-simulated datasets with changes from Conditioning to extinction learning phases estimated. (DOCX) [file pone.0268814.s001.docx]

| **Table S1.** *Conditioning – Extinction, N=30.* Strategy comparisons using Kendall rank correlation coefficient between effect-simulated datasets with changes from Conditioning to extinction learning phases estimated | | | | | |
| --- | --- | --- | --- | --- | --- |
|  |  | Strategy 1 | Strategy 2 | Strategy 3 | Strategy 4 |
| Strategy 1 | *_T_b* | 1 | 0.079 | 0.791 | -0.167 |
|  | Lower CI |  | 0.075 | 0.789 | -0.171 |
|  | Upper CI |  | 0.084 | 0.792 | -0.163 |
| Strategy 2 | *_T_b* |  | 1 | 0.025 | 0.002 |
|  | Lower CI |  |  | 0.021 | -0.002 |
|  | Upper CI |  |  | 0.030 | 0.006 |
| Strategy 3 | *_T_b* |  |  | 1 | -0.223 |
|  | Lower CI |  |  |  | -0.227 |
|  | Upper CI |  |  |  | -0.219 |
| Strategy 4 | *_T_b* |  |  |  | 1 |
|  | Lower CI |  |  |  |  |
|  | Upper CI |  |  |  |  |

**Supporting Information**

**Data where group-level effects were simulated**

**Conditioning - Extinction**
