# Supplementary material for: Methodological implications of sample size and extinction gradient on the robustness of fear conditioning across different analytic strategies
Source: PLoS One. 2022 May 24;17(5):e0268814. doi: 10.1371/journal.pone.0268814 (PMC9128987; doi:10.1371/journal.pone.0268814)
Supplement: S3 Table — Strategy comparisons using Kendall rank correlation coefficient between effect-simulated datasets with changes from Conditioning to extinction learning phases estimated. (DOCX) [file pone.0268814.s003.docx]

**Supporting Information**

**Data where group-level effects were simulated**

**Conditioning - Extinction**

| **Table S3.** *Conditioning – Extinction, N=120.* Strategy comparisons using Kendall rank correlation coefficient between effect-simulated datasets with changes from Conditioning to extinction learning phases estimated | | | | | |
| --- | --- | --- | --- | --- | --- |
|  |  | Strategy 1 | Strategy 2 | Strategy 3 | Strategy 4 |
| Strategy 1 | *_T_b* | 1 | -0.077 | 0.855 | -0.286 |
|  | Lower CI |  | -0.081 | 0.854 | -0.290 |
|  | Upper CI |  | -0.072 | 0.856 | -0.282 |
| Strategy 2 | *_T_b* |  | 1 | -0.065 | 0.000 |
|  | Lower CI |  |  | -0.070 | -0.004 |
|  | Upper CI |  |  | -0.061 | 0.005 |
| Strategy 3 | *_T_b* |  |  | 1 | -0.343 |
|  | Lower CI |  |  |  | -0.347 |
|  | Upper CI |  |  |  | -0.339 |
| Strategy 4 | *_T_b* |  |  |  | 1 |
|  | Lower CI |  |  |  |  |
|  | Upper CI |  |  |  |  |
