# Supplementary material for: Methodological implications of sample size and extinction gradient on the robustness of fear conditioning across different analytic strategies
Source: PLoS One. 2022 May 24;17(5):e0268814. doi: 10.1371/journal.pone.0268814 (PMC9128987; doi:10.1371/journal.pone.0268814)
Supplement: S8 Table — Strategy comparisons using Kendall rank correlation coefficient between effect-simulated datasets with a static extinction learning efficacy estimated. (DOCX) [file pone.0268814.s008.docx]

**Supporting Information**

**Data where group-level effects were simulated**

**Static Extinction**

| **Table S8.** *Static Extinction, N=30.* Strategy comparisons using Kendall rank correlation coefficient between effect-simulated datasets with a static extinction learning efficacy estimated | | | | | | | | |
| --- | --- | --- | --- | --- | --- | --- | --- | --- |
|  |  | Strategy 1 | Strategy 2 | Strategy 3 | Strategy 4 | Strategy 5 | Strategy 6 | Strategy 7 |
| Strategy 1 | *_T_b* | 1 | 0.071 | 0.591 | 0.013 | 0.062 | -0.056 | -0.031 |
|  | Lower CI |  | 0.067 | 0.589 | 0.008 | 0.057 | -0.060 | -0.035 |
|  | Upper CI |  | 0.075 | 0.594 | 0.017 | 0.066 | -0.052 | -0.027 |
| Strategy 2 | *_T_b* |  | 1 | 0.119 | 0.004 | -0.004 | 0.364 | 0.146 |
|  | Lower CI |  |  | 0.115 | 0.000 | -0.008 | 0.360 | 0.142 |
|  | Upper CI |  |  | 0.123 | 0.009 | -0.000 | 0.367 | 0.151 |
| Strategy 3 | *_T_b* |  |  | 1 | 0.038 | 0.109 | 0.001 | -0.000 |
|  | Lower CI |  |  |  | 0.033 | 0.105 | -0.002 | -0.004 |
|  | Upper CI |  |  |  | 0.042 | 0.114 | 0.005 | 0.003 |
| Strategy 4 | *_T_b* |  |  |  | 1 | 0.384 | 0.004 | 0.001 |
|  | Lower CI |  |  |  |  | 0.380 | 0.000 | -0.003 |
|  | Upper CI |  |  |  |  | 0.388 | 0.009 | 0.005 |
| Strategy 5 | *_T_b* |  |  |  |  | 1 | 0.002 | 0.001 |
|  | Lower CI |  |  |  |  |  | -0.001 | -0.003 |
|  | Upper CI |  |  |  |  |  | 0.006 | 0.005 |
| Strategy 6 | *_T_b* |  |  |  |  |  | 1 | 0.083 |
|  | Lower CI |  |  |  |  |  |  | 0.078 |
|  | Upper CI |  |  |  |  |  |  | 0.087 |
| Strategy 7 | *_T_b* |  |  |  |  |  |  | 1 |
|  | Lower CI |  |  |  |  |  |  |  |
|  | Upper CI |  |  |  |  |  |  |  |
