# Supplementary material for: Methodological implications of sample size and extinction gradient on the robustness of fear conditioning across different analytic strategies
Source: PLoS One. 2022 May 24;17(5):e0268814. doi: 10.1371/journal.pone.0268814 (PMC9128987; doi:10.1371/journal.pone.0268814)
Supplement: S9 Table — Strategy comparisons using Kendall rank correlation coefficient between effect-simulated datasets with a static extinction learning efficacy estimated. (DOCX) [file pone.0268814.s009.docx]

**Supporting Information**

**Data where group-level effects were simulated**

**Static Extinction**

| **Table S9.** *Static Extinction, N=60.* Strategy comparisons using Kendall rank correlation coefficient between effect-simulated datasets with a static extinction learning efficacy estimated | | | | | | | | |
| --- | --- | --- | --- | --- | --- | --- | --- | --- |
|  |  | Strategy 1 | Strategy 2 | Strategy 3 | Strategy 4 | Strategy 5 | Strategy 6 | Strategy 7 |
| Strategy 1 | *_T_b* | 1 | 0.066 | 0.624 | 0.000 | 0.067 | -0.061 | -0.029 |
|  | Lower CI |  | 0.061 | 0.622 | -0.003 | 0.062 | -0.066 | -0.033 |
|  | Upper CI |  | 0.070 | 0.626 | 0.005 | 0.072 | -0.057 | -0.025 |
| Strategy 2 | *_T_b* |  | 1 | 0.127 | 0.002 | -0.008 | 0.367 | 0.171 |
|  | Lower CI |  |  | 0.123 | -0.002 | -0.013 | 0.364 | 0.167 |
|  | Upper CI |  |  | 0.131 | 0.006 | -0.004 | 0.371 | 0.175 |
| Strategy 3 | *_T_b* |  |  | 1 | 0.003 | 0.091 | 0.001 | 0.000 |
|  | Lower CI |  |  |  | -0.001 | 0.086 | -0.002 | -0.003 |
|  | Upper CI |  |  |  | 0.007 | 0.096 | 0.005 | 0.004 |
| Strategy 4 | *_T_b* |  |  |  | 1 | 0.380 | 0.000 | -0.000 |
|  | Lower CI |  |  |  |  | 0.376 | -0.003 | -0.005 |
|  | Upper CI |  |  |  |  | 0.384 | 0.004 | 0.003 |
| Strategy 5 | *_T_b* |  |  |  |  | 1 | -0.000 | 0.000 |
|  | Lower CI |  |  |  |  |  | -0.004 | -0.003 |
|  | Upper CI |  |  |  |  |  | 0.003 | 0.004 |
| Strategy 6 | *_T_b* |  |  |  |  |  | 1 | 0.093 |
|  | Lower CI |  |  |  |  |  |  | 0.089 |
|  | Upper CI |  |  |  |  |  |  | 0.098 |
| Strategy 7 | *_T_b* |  |  |  |  |  |  | 1 |
|  | Lower CI |  |  |  |  |  |  |  |
|  | Upper CI |  |  |  |  |  |  |  |
