# Supplementary material for: Methodological implications of sample size and extinction gradient on the robustness of fear conditioning across different analytic strategies
Source: PLoS One. 2022 May 24;17(5):e0268814. doi: 10.1371/journal.pone.0268814 (PMC9128987; doi:10.1371/journal.pone.0268814)
Supplement: S10 Table — Strategy comparisons using Kendall rank correlation coefficient between effect-simulated datasets with a static extinction learning efficacy estimated. (DOCX) [file pone.0268814.s010.docx]

**Supporting Information**

**Data where group-level effects were simulated**

**Static Extinction**

| **Table S10.** *Static Extinction, N=120.* Strategy comparisons using Kendall rank correlation coefficient between effect-simulated datasets with a static extinction learning efficacy estimated | | | | | | | | |
| --- | --- | --- | --- | --- | --- | --- | --- | --- |
|  |  | Strategy 1 | Strategy 2 | Strategy 3 | Strategy 4 | Strategy 5 | Strategy 6 | Strategy 7 |
| Strategy 1 | *_T_b* | 1 | 0.067 | 0.631 | -0.000 | 0.095 | -0.060 | -0.031 |
|  | Lower CI |  | 0.063 | 0.629 | -0.004 | 0.090 | -0.064 | -0.035 |
|  | Upper CI |  | 0.071 | 0.634 | 0.004 | 0.100 | -0.056 | -0.026 |
| Strategy 2 | *_T_b* |  | 1 | 0.129 | 0.000 | -0.015 | 0.374 | 0.182 |
|  | Lower CI |  |  | 0.125 | -0.003 | -0.020 | 0.371 | 0.178 |
|  | Upper CI |  |  | 0.133 | 0.004 | -0.011 | 0.378 | 0.187 |
| Strategy 3 | *_T_b* |  |  | 1 | -0.000 | 0.122 | 0.000 | -0.001 |
|  | Lower CI |  |  |  | -0.004 | 0.117 | -0.004 | -0.005 |
|  | Upper CI |  |  |  | 0.004 | 0.127 | 0.004 | 0.002 |
| Strategy 4 | *_T_b* |  |  |  | 1 | 0.369 | 0.002 | -0.000 |
|  | Lower CI |  |  |  |  | 0.366 | -0.001 | -0.005 |
|  | Upper CI |  |  |  |  | 0.373 | 0.006 | 0.003 |
| Strategy 5 | *_T_b* |  |  |  |  | 1 | 0.003 | -0.000 |
|  | Lower CI |  |  |  |  |  | -0.001 | -0.004 |
|  | Upper CI |  |  |  |  |  | 0.007 | 0.003 |
| Strategy 6 | *_T_b* |  |  |  |  |  | 1 | 0.101 |
|  | Lower CI |  |  |  |  |  |  | 0.097 |
|  | Upper CI |  |  |  |  |  |  | 0.105 |
| Strategy 7 | *_T_b* |  |  |  |  |  |  | 1 |
|  | Lower CI |  |  |  |  |  |  |  |
|  | Upper CI |  |  |  |  |  |  |  |
