# Supplementary material for: Methodological implications of sample size and extinction gradient on the robustness of fear conditioning across different analytic strategies
Source: PLoS One. 2022 May 24;17(5):e0268814. doi: 10.1371/journal.pone.0268814 (PMC9128987; doi:10.1371/journal.pone.0268814)
Supplement: S11 Table — Strategy comparisons using Kendall rank correlation coefficient between effect-simulated datasets with a static extinction learning efficacy estimated. (DOCX) [file pone.0268814.s011.docx]

**Supporting Information**

**Data where group-level effects were simulated**

**Static Extinction**

| **Table S11.** *Static Extinction, N=240.* Strategy comparisons using Kendall rank correlation coefficient between effect-simulated datasets with a static extinction learning efficacy estimated | | | | | | | | |
| --- | --- | --- | --- | --- | --- | --- | --- | --- |
|  |  | Strategy 1 | Strategy 2 | Strategy 3 | Strategy 4 | Strategy 5 | Strategy 6 | Strategy 7 |
| Strategy 1 | *_T_b* | 1 | 0.065 | 0.631 | 0.001 | 0.139 | -0.062 | -0.026 |
|  | Lower CI |  | 0.061 | 0.629 | -0.002 | 0.134 | -0.066 | -0.030 |
|  | Upper CI |  | 0.069 | 0.634 | 0.006 | 0.144 | -0.058 | -0.022 |
| Strategy 2 | *_T_b* |  | 1 | 0.128 | 0.000 | -0.016 | 0.373 | 0.181 |
|  | Lower CI |  |  | 0.124 | -0.003 | -0.020 | 0.370 | 0.176 |
|  | Upper CI |  |  | 0.132 | 0.004 | -0.011 | 0.377 | 0.185 |
| Strategy 3 | *_T_b* |  |  | 1 | 0.001 | 0.177 | 0.000 | 0.005 |
|  | Lower CI |  |  |  | -0.003 | 0.172 | -0.003 | 0.001 |
|  | Upper CI |  |  |  | 0.006 | 0.182 | 0.004 | 0.009 |
| Strategy 4 | *_T_b* |  |  |  | 1 | 0.351 | 0.003 | -0.001 |
|  | Lower CI |  |  |  |  | 0.347 | -0.000 | -0.005 |
|  | Upper CI |  |  |  |  | 0.354 | 0.007 | 0.002 |
| Strategy 5 | *_T_b* |  |  |  |  | 1 | 0.004 | 0.000 |
|  | Lower CI |  |  |  |  |  | 0.000 | -0.003 |
|  | Upper CI |  |  |  |  |  | 0.008 | 0.004 |
| Strategy 6 | *_T_b* |  |  |  |  |  | 1 | 0.099 |
|  | Lower CI |  |  |  |  |  |  | 0.095 |
|  | Upper CI |  |  |  |  |  |  | 0.103 |
| Strategy 7 | *_T_b* |  |  |  |  |  |  | 1 |
|  | Lower CI |  |  |  |  |  |  |  |
|  | Upper CI |  |  |  |  |  |  |  |
