# Supplementary material for: Methodological implications of sample size and extinction gradient on the robustness of fear conditioning across different analytic strategies
Source: PLoS One. 2022 May 24;17(5):e0268814. doi: 10.1371/journal.pone.0268814 (PMC9128987; doi:10.1371/journal.pone.0268814)
Supplement: S12 Table — Strategy comparisons using Kendall rank correlation coefficient between effect-simulated datasets with a static extinction learning efficacy estimated. (DOCX) [file pone.0268814.s012.docx]

**Supporting Information**

**Data where group-level effects were simulated**

**Static Extinction**

| **Table S12.** *Static Extinction, N=480.* Strategy comparisons using Kendall rank correlation coefficient between effect-simulated datasets with a static extinction learning efficacy estimated | | | | | | | | |
| --- | --- | --- | --- | --- | --- | --- | --- | --- |
|  |  | Strategy 1 | Strategy 2 | Strategy 3 | Strategy 4 | Strategy 5 | Strategy 6 | Strategy 7 |
| Strategy 1 | *_T_b* | 1 | 0.066 | 0.634 | 0.002 | 0.193 | -0.058 | -0.032 |
|  | Lower CI |  | 0.062 | 0.631 | -0.002 | 0.189 | -0.062 | -0.036 |
|  | Upper CI |  | 0.071 | 0.636 | 0.007 | 0.198 | -0.054 | -0.027 |
| Strategy 2 | *_T_b* |  | 1 | 0.128 | -0.001 | -0.025 | 0.376 | 0.176 |
|  | Lower CI |  |  | 0.123 | -0.005 | -0.029 | 0.372 | 0.171 |
|  | Upper CI |  |  | 0.132 | 0.002 | -0.020 | 0.379 | 0.180 |
| Strategy 3 | *_T_b* |  |  | 1 | 0.000 | 0.243 | 0.002 | 0.000 |
|  | Lower CI |  |  |  | -0.004 | 0.238 | -0.001 | -0.003 |
|  | Upper CI |  |  |  | 0.005 | 0.248 | 0.006 | 0.004 |
| Strategy 4 | *_T_b* |  |  |  | 1 | 0.318 | -0.003 | 0.001 |
|  | Lower CI |  |  |  |  | 0.314 | -0.007 | -0.002 |
|  | Upper CI |  |  |  |  | 0.322 | 0.001 | 0.005 |
| Strategy 5 | *_T_b* |  |  |  |  | 1 | -0.000 | 0.002 |
|  | Lower CI |  |  |  |  |  | -0.005 | -0.001 |
|  | Upper CI |  |  |  |  |  | 0.003 | 0.006 |
| Strategy 6 | *_T_b* |  |  |  |  |  | 1 | 0.102 |
|  | Lower CI |  |  |  |  |  |  | 0.097 |
|  | Upper CI |  |  |  |  |  |  | 0.106 |
| Strategy 7 | *_T_b* |  |  |  |  |  |  | 1 |
|  | Lower CI |  |  |  |  |  |  |  |
|  | Upper CI |  |  |  |  |  |  |  |
