# Supplementary material for: Methodological implications of sample size and extinction gradient on the robustness of fear conditioning across different analytic strategies
Source: PLoS One. 2022 May 24;17(5):e0268814. doi: 10.1371/journal.pone.0268814 (PMC9128987; doi:10.1371/journal.pone.0268814)
Supplement: S13 Table — Strategy comparisons using Kendall rank correlation coefficient between effect-simulated datasets with a static extinction learning efficacy estimated. (DOCX) [file pone.0268814.s013.docx]

**Supporting Information**

**Data where group-level effects were simulated**

**Static Extinction**

| **Table S13.** *Static Extinction, N=720.* Strategy comparisons using Kendall rank correlation coefficient between effect-simulated datasets with a static extinction learning efficacy estimated | | | | | | | | |
| --- | --- | --- | --- | --- | --- | --- | --- | --- |
|  |  | Strategy 1 | Strategy 2 | Strategy 3 | Strategy 4 | Strategy 5 | Strategy 6 | Strategy 7 |
| Strategy 1 | *_T_b* | 1 | 0.066 | 0.633 | 0.002 | 0.228 | -0.060 | -0.033 |
|  | Lower CI |  | 0.062 | 0.631 | -0.002 | 0.224 | -0.065 | -0.037 |
|  | Upper CI |  | 0.071 | 0.635 | 0.006 | 0.233 | -0.056 | -0.028 |
| Strategy 2 | *_T_b* |  | 1 | 0.129 | 0.000 | -0.032 | 0.372 | 0.180 |
|  | Lower CI |  |  | 0.124 | -0.003 | -0.036 | 0.369 | 0.176 |
|  | Upper CI |  |  | 0.133 | 0.005 | -0.027 | 0.376 | 0.184 |
| Strategy 3 | *_T_b* |  |  | 1 | 0.002 | 0.289 | 0.001 | -0.001 |
|  | Lower CI |  |  |  | -0.002 | 0.284 | -0.003 | -0.005 |
|  | Upper CI |  |  |  | 0.007 | 0.293 | 0.005 | 0.003 |
| Strategy 4 | *_T_b* |  |  |  | 1 | 0.287 | -0.000 | -0.002 |
|  | Lower CI |  |  |  |  | 0.283 | -0.004 | -0.006 |
|  | Upper CI |  |  |  |  | 0.291 | 0.003 | 0.001 |
| Strategy 5 | *_T_b* |  |  |  |  | 1 | 0.001 | 0.000 |
|  | Lower CI |  |  |  |  |  | -0.003 | -0.004 |
|  | Upper CI |  |  |  |  |  | 0.005 | 0.004 |
| Strategy 6 | *_T_b* |  |  |  |  |  | 1 | 0.101 |
|  | Lower CI |  |  |  |  |  |  | 0.097 |
|  | Upper CI |  |  |  |  |  |  | 0.105 |
| Strategy 7 | *_T_b* |  |  |  |  |  |  | 1 |
|  | Lower CI |  |  |  |  |  |  |  |
|  | Upper CI |  |  |  |  |  |  |  |
