# Supplementary material for: Methodological implications of sample size and extinction gradient on the robustness of fear conditioning across different analytic strategies
Source: PLoS One. 2022 May 24;17(5):e0268814. doi: 10.1371/journal.pone.0268814 (PMC9128987; doi:10.1371/journal.pone.0268814)
Supplement: S15 Table — Strategy comparisons using Kendall rank correlation coefficient between effect-simulated datasets with changes during extinction learning estimated. (DOCX) [file pone.0268814.s015.docx]

**Supporting Information**

**Data where group-level effects were simulated**

**Early – Late Extinction**

| **Table S15.** *Early – Late Extinction, N=30.* Strategy comparisons using Kendall rank correlation coefficient between effect-simulated datasets with changes during extinction learning estimated | | | | | |
| --- | --- | --- | --- | --- | --- |
|  |  | Strategy 1 | Strategy 2 | Strategy 3 | Strategy 4 |
| Strategy 1 | *_T_b* | 1 | 0.159 | 0.171 | 0.141 |
|  | Lower CI |  | 0.155 | 0.167 | 0.137 |
|  | Upper CI |  | 0.163 | 0.175 | 0.145 |
| Strategy 2 | *_T_b* |  | 1 | 0.331 | 0.461 |
|  | Lower CI |  |  | 0.327 | 0.458 |
|  | Upper CI |  |  | 0.334 | 0.465 |
| Strategy 3 | *_T_b* |  |  | 1 | 0.517 |
|  | Lower CI |  |  |  | 0.514 |
|  | Upper CI |  |  |  | 0.520 |
| Strategy 4 | *_T_b* |  |  |  | 1 |
|  | Lower CI |  |  |  |  |
|  | Upper CI |  |  |  |  |
