# Supplementary material for: Methodological implications of sample size and extinction gradient on the robustness of fear conditioning across different analytic strategies
Source: PLoS One. 2022 May 24;17(5):e0268814. doi: 10.1371/journal.pone.0268814 (PMC9128987; doi:10.1371/journal.pone.0268814)
Supplement: S20 Table — Strategy comparisons using Kendall rank correlation coefficient between effect-simulated datasets with changes during extinction learning estimated. (DOCX) [file pone.0268814.s020.docx]

**Supporting Information**

**Data where group-level effects were simulated**

**Early – Late Extinction**

| **Table S20.** *Early – Late Extinction, N=720.* Strategy comparisons using Kendall rank correlation coefficient between effect-simulated datasets with changes during extinction learning estimated | | | | | |
| --- | --- | --- | --- | --- | --- |
|  |  | Strategy 1 | Strategy 2 | Strategy 3 | Strategy 4 |
| Strategy 1 | *_T_b* | 1 | 0.228 | 0.286 | 0.220 |
|  | Lower CI |  | 0.224 | 0.282 | 0.216 |
|  | Upper CI |  | 0.232 | 0.289 | 0.224 |
| Strategy 2 | *_T_b* |  | 1 | 0.513 | 0.598 |
|  | Lower CI |  |  | 0.510 | 0.595 |
|  | Upper CI |  |  | 0.516 | 0.600 |
| Strategy 3 | *_T_b* |  |  | 1 | 0.705 |
|  | Lower CI |  |  |  | 0.703 |
|  | Upper CI |  |  |  | 0.707 |
| Strategy 4 | *_T_b* |  |  |  | 1 |
|  | Lower CI |  |  |  |  |
|  | Upper CI |  |  |  |  |
