# Supplementary material for: Methodological implications of sample size and extinction gradient on the robustness of fear conditioning across different analytic strategies
Source: PLoS One. 2022 May 24;17(5):e0268814. doi: 10.1371/journal.pone.0268814 (PMC9128987; doi:10.1371/journal.pone.0268814)
Supplement: S21 Table — Strategy comparisons using Kendall rank correlation coefficient between effect-simulated datasets with changes during extinction learning estimated. (DOCX) [file pone.0268814.s021.docx]

**Supporting Information**

**Data where group-level effects were simulated**

**Early – Late Extinction**

| **Table S21.** *Early – Late Extinction, N=960.* Strategy comparisons using Kendall rank correlation coefficient between effect-simulated datasets with changes during extinction learning estimated | | | | | |
| --- | --- | --- | --- | --- | --- |
|  |  | Strategy 1 | Strategy 2 | Strategy 3 | Strategy 4 |
| Strategy 1 | *_T_b* | 1 | 0.232 | 0.291 | 0.224 |
|  | Lower CI |  | 0.228 | 0.287 | 0.220 |
|  | Upper CI |  | 0.236 | 0.295 | 0.228 |
| Strategy 2 | *_T_b* |  | 1 | 0.514 | 0.599 |
|  | Lower CI |  |  | 0.511 | 0.597 |
|  | Upper CI |  |  | 0.517 | 0.602 |
| Strategy 3 | *_T_b* |  |  | 1 | 0.706 |
|  | Lower CI |  |  |  | 0.704 |
|  | Upper CI |  |  |  | 0.708 |
| Strategy 4 | *_T_b* |  |  |  | 1 |
|  | Lower CI |  |  |  |  |
|  | Upper CI |  |  |  |  |
