# Supplementary material for: Methodological implications of sample size and extinction gradient on the robustness of fear conditioning across different analytic strategies
Source: PLoS One. 2022 May 24;17(5):e0268814. doi: 10.1371/journal.pone.0268814 (PMC9128987; doi:10.1371/journal.pone.0268814)
Supplement: S24 Table — Strategy comparisons using Kendall rank correlation coefficient between datasets with changes from Conditioning to extinction learning phases estimated. (DOCX) [file pone.0268814.s024.docx]

**Supporting Information**

**Data where no group-level effects were expected**

**Conditioning - extinction**

| **Table S24.** *Conditioning – Extinction, N=120.* Strategy comparisons using Kendall rank correlation coefficient between datasets with changes from Conditioning to extinction learning phases estimated | | | | | |
| --- | --- | --- | --- | --- | --- |
|  |  | Strategy 1 | Strategy 2 | Strategy 3 | Strategy 4 |
| Strategy 1 | *_T_b* | 1 | 0.247 | 0.765 | 0.108 |
|  | Lower CI |  | 0.243 | 0.763 | 0.103 |
|  | Upper CI |  | 0.251 | 0.766 | 0.112 |
| Strategy 2 | *_T_b* |  | 1 | 0.138 | 0.001 |
|  | Lower CI |  |  | 0.134 | -0.002 |
|  | Upper CI |  |  | 0.142 | 0.005 |
| Strategy 3 | *_T_b* |  |  | 1 | 0.151 |
|  | Lower CI |  |  |  | 0.147 |
|  | Upper CI |  |  |  | 0.155 |
| Strategy 4 | *_T_b* |  |  |  | 1 |
|  | Lower CI |  |  |  |  |
|  | Upper CI |  |  |  |  |
