# Supplementary material for: Methodological implications of sample size and extinction gradient on the robustness of fear conditioning across different analytic strategies
Source: PLoS One. 2022 May 24;17(5):e0268814. doi: 10.1371/journal.pone.0268814 (PMC9128987; doi:10.1371/journal.pone.0268814)
Supplement: S30 Table — Strategy comparisons using Kendall rank correlation coefficient between datasets with a static extinction learning efficacy estimated. (DOCX) [file pone.0268814.s030.docx]

**Supporting Information**

**Data where no group-level effects were expected**

**Static Extinction**

| **Table S30.** *Static Extinction, N=60.* Strategy comparisons using Kendall rank correlation coefficient between datasets with a static extinction learning efficacy estimated | | | | | | | | |
| --- | --- | --- | --- | --- | --- | --- | --- | --- |
|  |  | Strategy 1 | Strategy 2 | Strategy 3 | Strategy 4 | Strategy 5 | Strategy 6 | Strategy 7 |
| Strategy 1 | *_T_b* | 1 | 0.004 | 0.411 | 0.158 | 0.258 | 0.010 | 0.002 |
|  | Lower CI |  | 0.000 | 0.408 | 0.154 | 0.254 | 0.006 | -0.001 |
|  | Upper CI |  | 0.008 | 0.414 | 0.162 | 0.262 | 0.014 | 0.006 |
| Strategy 2 | *_T_b* |  | 1 | 0.006 | 0.032 | 0.010 | 0.156 | 0.107 |
|  | Lower CI |  |  | 0.002 | 0.028 | 0.006 | 0.152 | 0.103 |
|  | Upper CI |  |  | 0.010 | 0.036 | 0.014 | 0.160 | 0.111 |
| Strategy 3 | *_T_b* |  |  | 1 | 0.239 | 0.414 | -0.000 | 0.000 |
|  | Lower CI |  |  |  | 0.235 | 0.411 | -0.004 | -0.003 |
|  | Upper CI |  |  |  | 0.243 | 0.418 | 0.003 | 0.004 |
| Strategy 4 | *_T_b* |  |  |  | 1 | 0.394 | -0.000 | 0.003 |
|  | Lower CI |  |  |  |  | 0.391 | -0.004 | -0.001 |
|  | Upper CI |  |  |  |  | 0.398 | 0.003 | 0.007 |
| Strategy 5 | *_T_b* |  |  |  |  | 1 | -0.001 | -0.001 |
|  | Lower CI |  |  |  |  |  | -0.005 | -0.005 |
|  | Upper CI |  |  |  |  |  | 0.002 | 0.003 |
| Strategy 6 | *_T_b* |  |  |  |  |  | 1 | 0.135 |
|  | Lower CI |  |  |  |  |  |  | 0.131 |
|  | Upper CI |  |  |  |  |  |  | 0.139 |
| Strategy 7 | *_T_b* |  |  |  |  |  |  | 1 |
|  | Lower CI |  |  |  |  |  |  |  |
|  | Upper CI |  |  |  |  |  |  |  |
