# Supplementary material for: Methodological implications of sample size and extinction gradient on the robustness of fear conditioning across different analytic strategies
Source: PLoS One. 2022 May 24;17(5):e0268814. doi: 10.1371/journal.pone.0268814 (PMC9128987; doi:10.1371/journal.pone.0268814)
Supplement: S31 Table — Strategy comparisons using Kendall rank correlation coefficient between datasets with a static extinction learning efficacy estimated. (DOCX) [file pone.0268814.s031.docx]

**Supporting Information**

**Data where no group-level effects were expected**

**Static Extinction**

| **Table S31.** *Static Extinction, N=120.* Strategy comparisons using Kendall rank correlation coefficient between datasets with a static extinction learning efficacy estimated | | | | | | | | |
| --- | --- | --- | --- | --- | --- | --- | --- | --- |
|  |  | Strategy 1 | Strategy 2 | Strategy 3 | Strategy 4 | Strategy 5 | Strategy 6 | Strategy 7 |
| Strategy 1 | *_T_b* | 1 | 0.000 | 0.407 | 0.157 | 0.259 | 0.005 | -0.000 |
|  | Lower CI |  | -0.003 | 0.404 | 0.153 | 0.255 | 0.001 | -0.004 |
|  | Upper CI |  | 0.004 | 0.411 | 0.161 | 0.263 | 0.009 | 0.003 |
| Strategy 2 | *_T_b* |  | 1 | 0.004 | 0.030 | 0.012 | 0.157 | 0.105 |
|  | Lower CI |  |  | 0.000 | 0.025 | 0.007 | 0.153 | 0.101 |
|  | Upper CI |  |  | 0.008 | 0.034 | 0.016 | 0.161 | 0.109 |
| Strategy 3 | *_T_b* |  |  | 1 | 0.236 | 0.416 | -0.000 | 0.002 |
|  | Lower CI |  |  |  | 0.232 | 0.412 | -0.004 | -0.001 |
|  | Upper CI |  |  |  | 0.240 | 0.419 | 0.004 | 0.006 |
| Strategy 4 | *_T_b* |  |  |  | 1 | 0.397 | -0.000 | 0.003 |
|  | Lower CI |  |  |  |  | 0.394 | -0.004 | -0.000 |
|  | Upper CI |  |  |  |  | 0.401 | 0.003 | 0.007 |
| Strategy 5 | *_T_b* |  |  |  |  | 1 | -0.001 | 0.002 |
|  | Lower CI |  |  |  |  |  | -0.005 | -0.001 |
|  | Upper CI |  |  |  |  |  | 0.002 | 0.006 |
| Strategy 6 | *_T_b* |  |  |  |  |  | 1 | 0.129 |
|  | Lower CI |  |  |  |  |  |  | 0.125 |
|  | Upper CI |  |  |  |  |  |  | 0.133 |
| Strategy 7 | *_T_b* |  |  |  |  |  |  | 1 |
|  | Lower CI |  |  |  |  |  |  |  |
|  | Upper CI |  |  |  |  |  |  |  |
