# Supplementary material for: Methodological implications of sample size and extinction gradient on the robustness of fear conditioning across different analytic strategies
Source: PLoS One. 2022 May 24;17(5):e0268814. doi: 10.1371/journal.pone.0268814 (PMC9128987; doi:10.1371/journal.pone.0268814)
Supplement: S32 Table — Strategy comparisons using Kendall rank correlation coefficient between datasets with a static extinction learning efficacy estimated. (DOCX) [file pone.0268814.s032.docx]

**Supporting Information**

**Data where no group-level effects were expected**

**Static Extinction**

| **Table S32.** *Static Extinction, N=240.* Strategy comparisons using Kendall rank correlation coefficient between datasets with a static extinction learning efficacy estimated | | | | | | | | |
| --- | --- | --- | --- | --- | --- | --- | --- | --- |
|  |  | Strategy 1 | Strategy 2 | Strategy 3 | Strategy 4 | Strategy 5 | Strategy 6 | Strategy 7 |
| Strategy 1 | *_T_b* | 1 | 0.000 | 0.411 | 0.158 | 0.258 | 0.009 | -0.000 |
|  | Lower CI |  | -0.003 | 0.408 | 0.154 | 0.254 | 0.005 | -0.004 |
|  | Upper CI |  | 0.004 | 0.415 | 0.162 | 0.262 | 0.013 | 0.003 |
| Strategy 2 | *_T_b* |  | 1 | 0.005 | 0.028 | 0.012 | 0.155 | 0.103 |
|  | Lower CI |  |  | 0.001 | 0.024 | 0.008 | 0.151 | 0.099 |
|  | Upper CI |  |  | 0.010 | 0.033 | 0.016 | 0.159 | 0.107 |
| Strategy 3 | *_T_b* |  |  | 1 | 0.237 | 0.413 | 0.002 | -0.002 |
|  | Lower CI |  |  |  | 0.233 | 0.409 | -0.001 | -0.007 |
|  | Upper CI |  |  |  | 0.241 | 0.416 | 0.006 | 0.001 |
| Strategy 4 | *_T_b* |  |  |  | 1 | 0.399 | 0.000 | 0.002 |
|  | Lower CI |  |  |  |  | 0.395 | -0.003 | -0.001 |
|  | Upper CI |  |  |  |  | 0.402 | 0.004 | 0.006 |
| Strategy 5 | *_T_b* |  |  |  |  | 1 | 0.000 | -0.001 |
|  | Lower CI |  |  |  |  |  | -0.004 | -0.005 |
|  | Upper CI |  |  |  |  |  | 0.004 | 0.002 |
| Strategy 6 | *_T_b* |  |  |  |  |  | 1 | 0.128 |
|  | Lower CI |  |  |  |  |  |  | 0.124 |
|  | Upper CI |  |  |  |  |  |  | 0.133 |
| Strategy 7 | *_T_b* |  |  |  |  |  |  | 1 |
|  | Lower CI |  |  |  |  |  |  |  |
|  | Upper CI |  |  |  |  |  |  |  |
