# Supplementary material for: Methodological implications of sample size and extinction gradient on the robustness of fear conditioning across different analytic strategies
Source: PLoS One. 2022 May 24;17(5):e0268814. doi: 10.1371/journal.pone.0268814 (PMC9128987; doi:10.1371/journal.pone.0268814)
Supplement: S33 Table — Strategy comparisons using Kendall rank correlation coefficient between datasets with a static extinction learning efficacy estimated. (DOCX) [file pone.0268814.s033.docx]

**Supporting Information**

**Data where no group-level effects were expected**

**Static Extinction**

| **Table S33.** *Static Extinction, N=480.* Strategy comparisons using Kendall rank correlation coefficient between datasets with a static extinction learning efficacy estimated | | | | | | | | |
| --- | --- | --- | --- | --- | --- | --- | --- | --- |
|  |  | Strategy 1 | Strategy 2 | Strategy 3 | Strategy 4 | Strategy 5 | Strategy 6 | Strategy 7 |
| Strategy 1 | *_T_b* | 1 | 0.021 | 0.405 | 0.167 | 0.261 | 0.012 | -0.004 |
|  | Lower CI |  | 0.017 | 0.402 | 0.162 | 0.257 | 0.008 | -0.008 |
|  | Upper CI |  | 0.026 | 0.409 | 0.171 | 0.265 | 0.016 | -0.000 |
| Strategy 2 | *_T_b* |  | 1 | 0.013 | 0.026 | 0.014 | 0.158 | 0.122 |
|  | Lower CI |  |  | 0.010 | 0.022 | 0.010 | 0.154 | 0.118 |
|  | Upper CI |  |  | 0.017 | 0.031 | 0.017 | 0.161 | 0.127 |
| Strategy 3 | *_T_b* |  |  | 1 | 0.243 | 0.419 | 0.000 | 0.006 |
|  | Lower CI |  |  |  | 0.239 | 0.416 | -0.003 | 0.001 |
|  | Upper CI |  |  |  | 0.246 | 0.423 | 0.004 | 0.010 |
| Strategy 4 | *_T_b* |  |  |  | 1 | 0.399 | -0.003 | 0.001 |
|  | Lower CI |  |  |  |  | 0.396 | -0.007 | -0.003 |
|  | Upper CI |  |  |  |  | 0.402 | 0.001 | 0.005 |
| Strategy 5 | *_T_b* |  |  |  |  | 1 | -0.002 | 0.003 |
|  | Lower CI |  |  |  |  |  | -0.006 | -0.000 |
|  | Upper CI |  |  |  |  |  | 0.002 | 0.008 |
| Strategy 6 | *_T_b* |  |  |  |  |  | 1 | 0.139 |
|  | Lower CI |  |  |  |  |  |  | 0.135 |
|  | Upper CI |  |  |  |  |  |  | 0.143 |
| Strategy 7 | *_T_b* |  |  |  |  |  |  | 1 |
|  | Lower CI |  |  |  |  |  |  |  |
|  | Upper CI |  |  |  |  |  |  |  |
