# Supplementary material for: Methodological implications of sample size and extinction gradient on the robustness of fear conditioning across different analytic strategies
Source: PLoS One. 2022 May 24;17(5):e0268814. doi: 10.1371/journal.pone.0268814 (PMC9128987; doi:10.1371/journal.pone.0268814)
Supplement: S37 Table — Strategy comparisons using Kendall rank correlation coefficient between datasets with changes during extinction learning estimated. (DOCX) [file pone.0268814.s037.docx]

**Supporting Information**

**Data where no group-level effects were expected**

**Early – Late Extinction**

| **Table S37.** *Early – Late Extinction, N=60.* Strategy comparisons using Kendall rank correlation coefficient between datasets with changes during extinction learning estimated | | | | | |
| --- | --- | --- | --- | --- | --- |
|  |  | Strategy 1 | Strategy 2 | Strategy 3 | Strategy 4 |
| Strategy 1 | *_T_b* | 1 | 0.144 | 0.073 | 0.079 |
|  | Lower CI |  | 0.139 | 0.069 | 0.075 |
|  | Upper CI |  | 0.148 | 0.077 | 0.083 |
| Strategy 2 | *_T_b* |  | 1 | 0.220 | 0.270 |
|  | Lower CI |  |  | 0.215 | 0.266 |
|  | Upper CI |  |  | 0.224 | 0.275 |
| Strategy 3 | *_T_b* |  |  | 1 | 0.555 |
|  | Lower CI |  |  |  | 0.552 |
|  | Upper CI |  |  |  | 0.558 |
| Strategy 4 | *_T_b* |  |  |  | 1 |
|  | Lower CI |  |  |  |  |
|  | Upper CI |  |  |  |  |
