# Supplementary material for: Methodological implications of sample size and extinction gradient on the robustness of fear conditioning across different analytic strategies
Source: PLoS One. 2022 May 24;17(5):e0268814. doi: 10.1371/journal.pone.0268814 (PMC9128987; doi:10.1371/journal.pone.0268814)
Supplement: S40 Table — Strategy comparisons using Kendall rank correlation coefficient between datasets with changes during extinction learning estimated. (DOCX) [file pone.0268814.s040.docx]

**Supporting Information**

**Data where no group-level effects were expected**

**Early – Late Extinction**

| **Table S40.** *Early – Late Extinction, N=480.* Strategy comparisons using Kendall rank correlation coefficient between datasets with changes during extinction learning estimated | | | | | |
| --- | --- | --- | --- | --- | --- |
|  |  | Strategy 1 | Strategy 2 | Strategy 3 | Strategy 4 |
| Strategy 1 | *_T_b* | 1 | 0.147 | 0.085 | 0.089 |
|  | Lower CI |  | 0.143 | 0.081 | 0.085 |
|  | Upper CI |  | 0.150 | 0.090 | 0.093 |
| Strategy 2 | *_T_b* |  | 1 | 0.234 | 0.284 |
|  | Lower CI |  |  | 0.230 | 0.280 |
|  | Upper CI |  |  | 0.238 | 0.288 |
| Strategy 3 | *_T_b* |  |  | 1 | 0.563 |
|  | Lower CI |  |  |  | 0.560 |
|  | Upper CI |  |  |  | 0.565 |
| Strategy 4 | *_T_b* |  |  |  | 1 |
|  | Lower CI |  |  |  |  |
|  | Upper CI |  |  |  |  |
